# Can ultrasonography be used to assess capsular distention in the painful temporomandibular joint?

**DOI:** 10.1186/s12903-021-01853-0

**Published:** 2021-10-06

**Authors:** Ji-Hoi Kim, Jung-Hyun Park, Jin-Woo Kim, Sun-Jong Kim

**Affiliations:** 1 Department of Oral Health Science, Ewha Womans University Graduate School of Clinical Dentistry , Seoul, Republic of Korea; 2 Present Address: Department of Orthodontics, Institute of Craniofacial Deformity, Yonsei University College of Dentistry, Seoul, Republic of Korea; 3 Department of Oral and Maxillofacial Surgery, College of Medicine, Ewha Womans University, Mok-Dong Hospital, 1071, Anyangcheon-ro, Yangcheon-gu, Seoul, 07985 Republic of Korea

**Keywords:** Temporomandibular joint, Temporomandibular disorder, Ultrasonography, Capsular width, Joint pain

## Abstract

**Background:**

To determine whether capsular distention in the painful temporomandibular joint (TMJ) can be assessed by ultrasonography, we compared the capsular width between painful TMJs and painless TMJ. The risk factors for TMJ pain were also investigated including capsular width and other clinical factors such as TMJ sounds that may affect the occurrence and persistence of TMJ pain.

**Methods:**

TMJ ultrasonography was performed on 87 temporomandibular disorder (TMD) patients, including 47 unilateral and 29 bilateral TMJ pain patients, and 11 patients without TMJ pain.

**Results:**

The capsular width was greater in the 105 painful joints than in the 69 painless joints. Considering individual anatomical variations, the differences between painful and painless joints in unilateral TMJ pain patients were also analyzed, revealing a greater width in painful joints. Capsular width was a risk factor for TMJ pain with an adjusted odds ratio of 1.496 (95% confidence interval 1.312–1.706; *p* < 0.001) and was significantly correlated with pain scores.

**Conclusion:**

This correlation may suggest that pain intensity is associated with widened capsular width because of joint effusion or synovitis. Further studies are required to refine and establish the protocols for standard examinations using ultrasound imaging.

**Supplementary Information:**

The online version contains supplementary material available at 10.1186/s12903-021-01853-0.

## Background

The temporomandibular joint (TMJ) is a synovial joint consisting of the mandibular condyle, mandibular fossa, articular disc, and articular capsule. Temporomandibular disorder (TMD) is defined as the development of articular disc disorders, arthritis, dislocation, and masticatory disturbances owing to external and internal factors that affect the TMJ [1]. The symptoms of TMD include muscle and joint pain, TMJ sounds, and limitations in the mandibular movements, often accompanied by tinnitus and headache. Facial pain localized in the TMJ is often attributed to inflammation of the synovium, which causes synovial effusion [2, 3]. In general, panoramic radiography, computed tomography (CT), and magnetic resonance imaging (MRI) have been widely employed for diagnostic imaging examinations of the TMJ. Although panoramic radiography provides gross information on the TMJ anatomy, it has certain limitations as it generates single plane images with low clarity owing to superimposition and distortion. CT generates multiplanar images and is used as a standard modality for evaluating the TMJ’s bony structures; however, clear images of the soft tissue cannot be obtained with CT, and it is associated with high radiation exposure. MRI is often used for imaging examinations of the soft tissues; however, it is uneconomical, difficult to access, and a relatively long time is required for reaching diagnoses. Because of the limitation of imaging modalities of the TMJ, there has been a need for an alternative diagnostic tool for the clinical evaluation of TMD.

Ultrasonography is easily accessible and affordable, making it a viable and potent imaging modality for TMJ evaluation. Ultrasonography is a non-invasive, pain-free, economical, and real-time diagnostic modality that can be used in an outpatient setting. It has certain drawbacks, such as its limited access to deep structures or to articular discs owing to the absorption of the sound waves by the mandibular and temporal bones, as a result of which images of the internal tissue cannot be properly formed. However, by appropriately adjusting the position of the probe, images of the articular space and capsule can be captured. The articular space has been evaluated in other joints, such as knee, elbow, hip, and shoulder in cases involving inflammatory joint effusion. Koski et al. evaluated the capsular width of the hip joints in patients with chronic inflammatory disease using ultrasonography. They demonstrated that the capsular width in synovitis patients was greater than the capsular width following treatment in arthritis synovitis patients [4]. Friedman et al. reported that ultrasonography of the knee can be applied clinically to assess knee tendons, muscles, and ligaments, as well as joint effusions and synovial thickening [5].

Further, the use of TMJ ultrasonography has been reported in determining the presence of TMJ inflammation by measuring the capsular width [6]. By studying ultrasonography measurements of the capsular width and MRI diagnostic data associated with TMJ effusion, Manfredini et al. reported that ultrasound assessment results indicating increased capsular width could be a predictor of TMJ effusion [7]. Bas et al. also suggested TMJ ultrasonography as a screening method based on joint effusion detected via MRI and increased capsular width via ultrasonography that was significantly associated with TMJ pain [8]. Though previous studies have values in uncovering ultrasonographic capsular width related to TMJ effusion, they only assessed MRI as a reference standard without considering clinical factors that could affect TMD development and the persistence of pain.

The aim of the study was to determine whether capsular distention in the painful TMJ can be assessed by ultrasonography. Based on the hypothesis that there is widening in capsular width induced from the joint effusion or synovial thickening which can cause TMJ pain, we compared the capsular width by ultrasonography between painful TMJs and painless TMJs. We not only compared average capsular widths similar to in previous studies, but we also analyzed the differences between painful joints and painless joints in unilateral TMJ pain patients considering individual anatomical variations. The risk factors for TMJ pain, including capsular width and other clinical factors that could affect TMD development and the persistence of pain, were investigated to evaluate the associations.

## Methods

### Clinical evaluations

This study was approved by the Institutional Bioethics Review Board of Ewha Womans University, Mokdong Hospital (IRB No. 2019-02-036), and it complies with the most recent Helsinki Declaration revised in 2013. Inclusion criteria were patients who were diagnosed as TMD in the Department of Oral and Maxillofacial Surgery of Ewha Womans University, Mokdong Hospital, from 1 June 2017 to 31 January 2019. The diagnosis was established according to the Research Diagnostic Criteria for Temporomandibular Disorder (RDC/TMD) diagnostic decision tree based on patient’s history and comprehensive clinical examination [9, 10]. Panoramic and tanscranial radiographs evaluated all patients to assess gross anatomical and functional abnormality in the TMJ and other jaw areas. Clinical examination of TMJs and surrounding muscles, including masticatory and neck muscles was performed. The clinical examination included palpation of the TMJs and muscles, assessment of a range of jaw movement, assessment of pain characteristics at rest, function and clenching, assessment of intraoral condition, and examination questionnaire including history of jaw locking, psychological and behavioral factors. Exclusion criteria were patients with histories of hypersensitivity to ultrasound gels or of condylar fractures and those who could not produce records of ultrasound images or clinical examinations were excluded from the study. A total of 87 patients with TMD was included in this study.

According to the localized TMJ pain, the 87 included patients were assigned to either the TMJ pain group (n = 76) or the painless group (n = 11). There were no patients who had pain localized in the TMJ area in the painless group. Among 11 patients in the painless group, 8 patients only had TMJ sounds without other symptoms, and 4 patients had myalgia in the neck area. The pain group consisted of 29 patients with bilateral TMJ pain and 47 patients with unilateral TMJ pain. In terms of the number of TMJs in total, 174 bilateral TMJs evaluated in 87 patients including 105 TMJs in the painful joint group and 69 TMJs in the painless joint group.

Clinical characteristics such as patient sex, age, history of orthodontic treatment, TMJ sounds, pain duration, pain score, and maximum mouth opening were collected. TMJ sounds were examined by palpation on both TMJ during mouth opening and closing. Pain scores were measured using a numerical rating scale (NRS), and patients were asked to assign a score to their pain during mouth opening within the range of 0–10. The maximum mouth opening was evaluated by measuring the distance between the central incisors of the maxilla and mandible in millimeters (mm) when the patient’s mouth was maximally open. Oral conditions including tooth attrition, tongue ridging, and buccal mucosa ridging were examined. Tooth attrition was defined as 2–4 degree of wear on the incisal edges and cusp tips according to the Smith and Knight Tooth Wear Index [11]. Based on a previous report, buccal mucosa ridging was defined as linear thickening where the teeth occlude on the buccal mucosa [12]. Tongue ridging was defined as a scalloped lateral margin of the tongue.

Data on psychological and behavioral factors that could affect TMD development and the persistence of pain were also collected. The psychological factors included the presence of stress, depression, and anxiety. The behavioral factors included bruxism, clenching, side sleep, unilateral chewing, chin leaning, alcohol consumption, caffeine consumption, snoring, and forward head posture. Questionnaires were used to assess psychological and behavioral factors (Additional file 1: Table S1) [13]. Craniovertebral angle less than 50° was used to determine forward head posture [14, 15].

### Ultrasonography evaluations

**Fig. 1:**
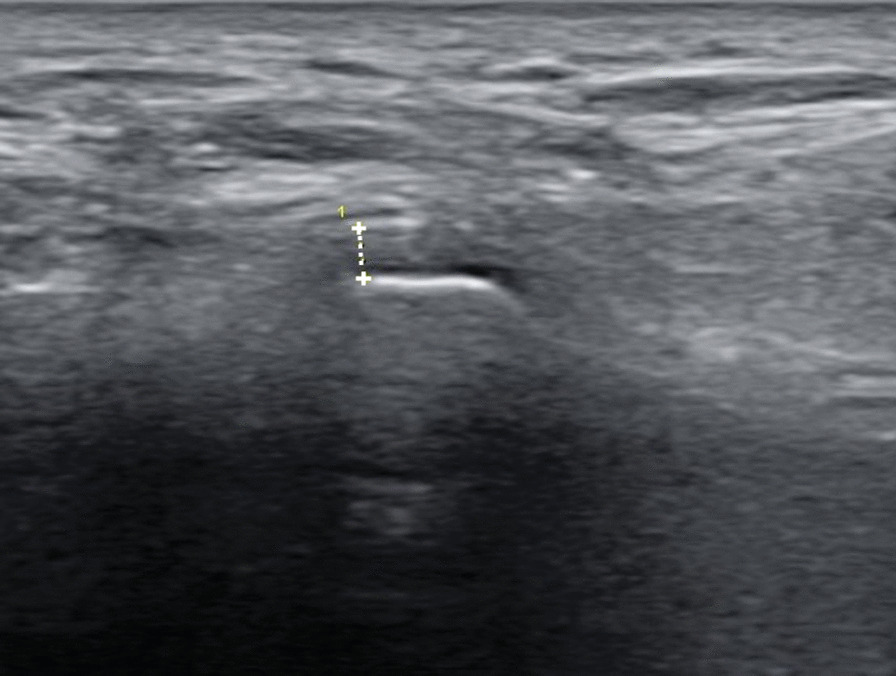
Measurement of capsular width; distance between the articular capsule and condylar superolateral surface in the closed mouth position. Upper + , articular capsule; lower + , mandibular condyle

The ultrasound equipment used consisted of an E-CUBE 9 Diamond scanner (Alpinion medical systems®, Seoul, Korea) and a 3–12 MHz linear probe. TMJ ultrasonography was performed by a single, trained examiner blinded on clinical findings. Intraclass correlation coefficient (ICC) was estimated to evaluate intra-examiner reliability. Obtained ICC value (95% confidence interval) was 0.971 (0.661–0.991), which indicates the level of reliability was moderate to excellent [16]. The patients were seated with their backs reclined. Before capturing an ultrasonography image, the opening and closure of each patient’s mouth were induced to detect the position of TMJ. The linear probe was applied perpendicular to the zygomatic arch and tilted until an appropriate visual field was obtained. To ensure no pressure was applied on the capsular width, the probe was positioned just above the skin surface without any skin depression. Hyperechoic mandibular condyles and mandibular fossae were identified. The articular capsule was identified as a hyperechoic line running parallel to the surface of the mandibular condyle. Capsular width was measured as the distance between the articular capsule and condylar superolateral surface in the closed mouth position [7, 8]. The RadiAnt DICOM Viewer (Medixant, Poznan, Poland) software was used to measure the width with an accuracy of up to 0.1 mm (Fig. 1).

### Statistical analysis

The assumptions of normality and homogeneity of variance were confirmed using the Kolmogorov–Smirnov test before further statistical analyses. An independent t-test and a paired t-test were used to compare capsular widths. To identify the risk factors of TMJ pain, univariate and multivariate logistic regression analyses were used. The correlations between pain scores and capsular width and maximum mouth opening values were analyzed using Spearman correlation coefficient analysis. The statistically significant level was determined to be *p* < 0.05. The statistical analysis was performed using the software program, SPSS version 25.0 (IBM Corp., Armonk, NY, USA).

## Results

### Characteristics of patients

The clinical characteristics of the 87 patients included in this study are presented in Table 1. There were 27 males (31%) and 60 females (69%). The mean age was 39.5 ± 15.5 years, ranging from 16 to 82 years. Of the total 174 joints, 82 joints (47%) produced joint sounds, and 92 joints (53%) did not produce any sound. Further, 69 joints (39.7%) were painless (NRS 0), 38 joints (21.3%) had mild pain (NRS 1–3), 60 joints (34.5%) had moderate pain (NRS 4–6), and 7 joints (4%) had severe pain (NRS 7–10). The maximum mouth opening ranged from a minimum of 12 mm to a maximum of 58 mm. There were overlapping presentations in terms of oral conditions and psychological and behavioral factors among the subjects.

**Table1:** Clinical characteristics of the 87 patients and 174 joints included in this study

Clinical characteristics	No. of patients (%)
*Gender*	
Male	27 (31%)
Female	60 (69%)
Age, year (range)	39.5 ± 15.5 (16–82)
History of orthodontic treatment	9 (10.3%)
*Joint sound (n* = *174 joints)*	
( +)	82 (47%)
(−)	92 (53%)
*Pain duration (n* = *105 pain joint)*	
Less than 3 months	61 (58%)
More than 3 months	44 (42%)
*Pain score (n* = *174 joints)*	
None (NAS 0)	69 (39.7%)
Mild (NAS 1–3)	38 (21.8%)
Moderate (NAS 4–6)	60 (34.5%)
Severe (NAS 7–10)	7 (4%)
*Maximum mouth opening*	
Mean ± SD, mm (range)	37.2 ± 8.8 (12–58)
*Oral conditions*	
Tooth attrition	46 (52.9%)
Tongue ridging	44 (50.6%)
Buccal mucosa ridging	44 (50.6%)
*Psychological factors*	
Stress	32 (36.8%)
Depression	15 (17.2%)
Anxiety	17 (19.5%)
*Behavioral factors*	
Bruxism	12 (13.8%)
Clenching	29 (33.3%)
Side sleep	22 (25.3%)
Unilateral chewing	31 (35.6%)
Chin leaning	12 (13.8%)
Alcohol consumption	12 (13.8%)
Caffeine consumption	28 (32.2%)
Forward head posture	15 (17.2%)

### Relationship between capsular width and pain

Table 2 presents the mean capsular width in each group. The mean capsular widths of the 105 painful joints and 69 painless joints were 2.04 ± 0.49 mm and 1.37 ± 0.41 mm, respectively. The width was greater in the painful joints, and the difference in values between the two groups was statistically significant (*p* < 0.001). Concerning the anatomical variations in capsular widths, a paired comparison was performed for 47 unilateral pain patients, and the differences in capsular width were statistically significant (*p* < 0.001). The mean capsular width of the 47 painful joints was 2.04 ± 0.52 mm, and that of the 47 painless joints was 1.37 ± 0.36 mm. To determine the relationship between capsular width and pain duration, 105 painful joints were classified based on whether the pain was experienced for less than 3 months or for more than 3 months. The mean capsular width was not statistically different at 1.97 ± 0.45 mm for pain durations of less than 3 months and 2.15 ± 0.52 mm for pain durations of 3 months or more (*p* = 0.081).

**Table 2:** Relationship between capsular width assessed by ultrasonography and TMJ pain

	Capsular width (mm)	*p*-value
*Total patients (N* = *174 joints)*		
Pain ( +)	2.04 ± 0.49	< 0.001*
Pain ( −)	1.37 ± 0.41	
*Unilateral pain patients (N* = *47 paired joints)*		
Pain ( +)	2.04 ± 0.52	< 0.001*
Pain ( −)	1.37 ± 0.36	
*Pain duration*		
More than 3 months	2.15 ± 0.52	0.081
Less than 3 months	1.97 ± 0.45	

* Statistically significant by independent t-test and paired t-test

### Risk factors for TMJ pain

The associations between the TMJ pain and clinical factors were evaluated using logistic regression analysis. Based on the results of the univariate logistic regression analysis, capsular width, joint sounds, tooth attrition, and stress were significant risk factors for TMJ pain (Table 3). Adjusted odds ratios were obtained by multivariate logistic regression analysis with variables including sex, capsular width, joint sounds, tooth attrition, and stress (Table 4). We found that only capsular width was an independent risk factor for TMJ pain. The adjusted odds ratio for capsular width was 1.496 (95% confidence interval 1.312–1.706; *p* < 0.001), indicating that the risk of TMJ pain increased 1.496 times with a 0.1 mm increase in the capsular width. The correlation coefficient between the capsular width and pain score was 0.570 (*p* < 0.001) (Table 5). As the pain score increased, the capsular width tended to increase with a moderate level of correlation. Regarding the maximum mouth opening and pain score, the correlation coefficient was − 0.235, which implies that the maximum mouth opening decreased with increasing pain score (*p* = 0.002).

**Table 3:** Predictors for TMJ pain (unadjusted odds ratios)

Variables	Odds ratio	95% confidence interval	*P* value
Age	1.000	0.981–1.020	0.970
Sex†	0.852	0.440–1.652	0.636
Capsular width	1.497	1.132–1.697	< 0.001*
Joint sound	2.848	1.507–5.386	0.001*
Tooth attrition	2.069	1.116–3.835	0.021*
Tongue ridging	1.952	1.054–3.617	0.330
Buccal mucosa ridging	1.952	1.054–3.617	0.330
History of orthodontic treatment	1.809	0.614–5.324	0.282
Stress	2.209	1.140–4.280	0.019*
Depression	1.165	0.516–2.629	0.713
Anxiety	1.748	0.777–3.931	0.177
Bruxism	1.111	0.457–2.702	0.816
Clenching	1.555	0.804–3.009	0.190
Side sleep	1.571	0.762–3.240	0.221
Unilateral chewing	1.063	0.563–2.008	0.850
Chin leaning	2.172	0.816–5.784	0.120
Alcohol consumption	1.711	0.670–4.373	0.262
Caffeine consumption	1.143	0.594–2.198	0.689
Forward head posture	1.388	0.606–3.179	0.438

^†^Odds ratio of female relative to male

* Statistically significant by univariate logistic regression analysis

**Table 4:** Risk factors for TMJ pain (adjusted odds ratios)

Variables	Odds ratio	95% confidence interval	*P* value
Sex†	0.699	0.286–1.708	0.432
Capsular width	1.496	1.312–1.706	< 0.001*
Joint sound	1.451	0.633–3.323	0.379
Tooth attrition	1.126	0.464–2.732	0.793
Stress	2.294	0.897–5.865	0.083

^†^ Odds ratio of female relative to male

* Statistically significant by multivariate logistic regression analysis

**Table 5:** Correlation analysis between pain scores and capsular width and maximum mouth opening

	Capsular width	Maximum mouth opening
rho	0.570	− 0.235
*p*-value	< 0.001*	0.002*

* Statistically significant by Spearman’s correlation coefficient

## Discussion

In this study, capsular distention of TMJ was assessed using ultrasonography. This study suggests that the capsular width was greater in the painful joints than painless ones. Additionally, when evaluating the associations between the TMJ pain and clinical factors, distended capsular width may be an independent risk factor for TMJ pain. Most studies on TMJ ultrasonography have focused on detecting of disc displacement, and very few works have investigated capsular width measured using ultrasonography [17–19]. However, the present study showed the potential role of ultrasonography that can be used to assess capsular distention in the painful TMJ.

Manfredini et al. reported that ultrasound measurement of the capsular width could be an indirect marker for TMJ effusion [7]. Using receiver operating characteristic (ROC) curve analysis to assess the cutoff value for capsular width that could be used to discriminate among joints with and without an MRI diagnosis of effusion, they demonstrated that the cutoff value was around 2 mm. Bas et al. evaluated the relationship between MRI-diagnosed joint effusion, ultrasonography-evaluated capsular width, and TMJ pain. They reported that the cutoff value for capsular width evaluated using an ROC curve was found to be 1.65 mm [8]. They demonstrated that a significant positive correlation was found between the clinical pain scores and ultrasonographic capsular width and that the average pain score was lower in patients with capsular widths of less than the cutoff value (1.65 mm).

In the present study, the mean capsular width of the painful joints was 2.04 ± 0.49 mm, and that of the painless joints was 1.37 ± 0.41 mm, thereby exhibiting statistically significant differences. These results are similar to the results indicating the 2 mm capsular width threshold associated with joint effusion reported by Manfredini et al. [7]. In the present study, to determine whether individual anatomical variations affect the capsular width of painful or painless TMJs, the differences between the parameters of the painful and painless joints in the unilateral TMJ pain patients were also analyzed. A paired comparison in 47 unilateral pain patients revealed statistically significant differences in capsular widths. The mean capsular width of the 47 painful joints was 2.04 ± 0.52 mm, and that of the 47 painless joints was 1.37 ± 0.36 mm. The study demonstrated that capsular distention in painful TMJ resulting from joint effusion or synovial thickening may be assessed by TMJ ultrasonography. Because the differences in width were only 0.67 mm in average, cautions are needed to position the ultrasound probe on the TMJ and measure the width. To ensure no pressure is applied on the capsular width, the probe has to be positioned just above the skin surface, and multiple measuring of the width may be recommended.

To determine the risk factors for TMJ pain, the associations between TMJ pain and clinical factors including psychological and behavioral factors were evaluated. On our knowledge, this study is the first study estimating risk of capsular width on TMJ pain considering other clinical factors that could play a role in the occurrence and persistence of pain. Psychological and behavioral factors have been suggested as potential causes of TMD. Psychosocial factors, including depression, stress, and anxiety, may play a role in the onset and progression of TMD [13]. According to a study by Slade el al., depression, stress, and mood disorders were associated with pain sensitivity and were predictive of 2- to threefold increases in TMD risk [20]. The relationships between TMD and behavioral factors, such as clenching and bruxism, have also been reported; these factors cause the overload of the TMJ and masticatory muscles, and affecting the onset, persistence, and aggravation of TMD [13].

In the present study, capsular width, joint sounds, tooth attrition, and stress were found to be significant risk factors for TMJ pain in a univariate logistic regression analysis. In a multivariate analysis, however, only capsular width was an independent risk factor for TMJ pain. Further, the correlation between the capsular width and pain score revealed that the capsular width increased significantly with increasing pain score. This correlation may suggest that pain intensity that reflects the severity of joint space inflammation is associated with widened capsular width because of joint effusion or synovitis. It may also suggest that measuring capsular width in painful TMJ may be used to measure severity of joint inflammation and follow-up on the responses to treatment by measuring width decreases. If the width were reduced, it would imply that current treatment has resolved the inflammation in the TMJ. Because ultrasonography is a simple method that can be used in an outpatient clinic, the progress of treatment can be easily monitored using TMJ ultrasonography.

Johnston et al. assessed the association between TMJ inflammation as measured by ultrasound and patient disability assessed by the Steigerwald Maher TMD Disability Index (SMTDI). The authors reported that greater capsular widths were found to be significant predictors of SMTDI scores [3]. Although the relationship between functional disability of mandible and capsular width has not been determined in this study, both maximum mouth opening and capsular width significantly correlated with pain scores. Considering the negative correlation between joint pain and maximum mouth opening, measuring capsular width can be used as a potential approach to examine the disability of TMD patients. As evidence for symptoms, the use of maximum mouth opening is limited as it is measured via a patient-dependent examination; therefore, it is not technically an objective parameter, and measuring it can induce pain during examinations. As an alternative, measuring capsular width may be used as a potential indirect method to examine whether there was a disability of mandible.

This study has a limitation in that ultrasonography was not compared with MRI that is considered the standard imaging modality to validate evidence of effusion or synovitis. However, the results indicating that capsular distention in painful TMJ resulting from joint effusion or synovial thickening can be detected and evaluated by a simple ultrasonography technique is valuable. TMJ ultrasonography has not yet been established as a standard modality, and the accuracy of the results mainly depends on the operator’s training [18]. Further studies are still required to refine and establish the protocols for standard examinations using ultrasound imaging [21]. It is believed that the value of TMJ ultrasonography, a simple and inexpensive modality, can be recognized with the diagnostic standardization of the approach [17].

## Conclusion

The average capsular width of a painful TMJ was significantly greater than that of a painless TMJ. The capsular width increased significantly with increasing pain score. This correlation may suggest that pain intensity that reflects the severity of joint space inflammation is associated with widened capsular width because of joint effusion or synovitis. Further studies are required to refine and establish the protocols for standard examinations using ultrasound imaging.

## Supplementary Information

**Additional file 1. Table S1. Questionnaire.**

## Data Availability

All data are available from the corresponding author upon reasonable request.

## Abbreviations

TMJ: Temporomandibular joint
TMD: Temporomandibular disorder
CT: Computed tomography
MRI: Magnetic resonance imaging
NRS: Numerical rating scale
ICC: Intraclass correlation coefficient
ROC: Receiver operating characteristic
SMTDI: Steigerwald Maher TMD Disability Index
