# Supplementary material for: Can ultrasonography be used to assess capsular distention in the painful temporomandibular joint?
Source: BMC Oral Health. 2021 Oct 6;21:497. doi: 10.1186/s12903-021-01853-0 (PMC8493706; doi:10.1186/s12903-021-01853-0)
Supplement: Supplementary file 1 — Additional file 1. Table S1. Questionnaire. [file 12903_2021_1853_MOESM1_ESM.docx]

**Can ultrasonography be used to assess capsular distention in the painful temporomandibular joint?**

**Ji-Hoi Kim ^1^, Jung-Hyun Park ^2,^*, Jin-Woo Kim ^2^ and Sun-Jong Kim ^2^**

^1^Graduate, Department of Oral Health Science, Ewha Womans University Graduate School of Clinical Dentistry, Seoul, Republic of Korea

E-mail: jihoi3537@gmail.com

^2^Clinical Assistant Professor, Department of Oral and Maxillofacial surgery, College of Medicine, Ewha Womans University, Seoul, Republic of Korea

E-mail: omspark07@gmail.com

^3^Assistant Professor, Department of Oral and Maxillofacial surgery, College of Medicine, Ewha Womans University, Seoul, Republic of Korea

E-mail: jinu600@gmail.com

^4^Professor, Department of Oral and Maxillofacial surgery, College of Medicine, Ewha Womans University, Seoul, Republic of Korea

E-mail: sjsj7777@ewha.ac.kr

***Corresponding Author**

Jung-Hyun Park, DDS PhD

Clinical Assistant Professor, Department of Oral and Maxillofacial Surgery,

Ewha Womans University, Mok-dong Hospital

1071, Anyangcheon-ro, Yangcheon-gu, Seoul, 07985, Republic of Korea

Tel: 82-2-2650-2720 (clinic), Fax: 82-2-2650-2754

E-mail: [omspark07@gmail.com](mailto:omspark07@gmail.com)

Table S1. Questionnaire to assess psychological and behavioral factors.

| **Factors** | **Questions** |
| --- | --- |
| Stress | Do you experience stress at work, school, home, or in relationships? |
| Depression | Do you feel depressed now? |
| Anxiety | Do you experience anxiety at work, school, home, or in relationships? |
| Bruxism | Do you have a recent history of noises associated with nocturnal tooth grinding as reported by a third person? |
| Clenching | Do you often allow your upper- and lower teeth to make continuous contact during work or at rest? |
| Side sleep | Do you usually sleep on your side? |
| Unilateral chewing | When you chew food, do you prefer one side (unilateral) chewing? |
| Chin leaning | Do you have a habit of leaning your chin during work or at rest? |
| Alcohol consumption | Do you consume alcohol once a week or more? |
| Caffeine consumption | Do you consume one or more cups of caffeine per day? |
| Snoring | Do you have a recent history of noises associated with snoring as reported by a third person? |
